# Effects of Exercise to Improve Cardiovascular Health

**DOI:** 10.3389/fcvm.2019.00069

**Published:** 2019-06-04

**Authors:** Kelsey Pinckard, Kedryn K. Baskin, Kristin I. Stanford

**Affiliations:** Department of Physiology and Cell Biology, Dorothy M. Davis Heart and Lung Research Institute, The Ohio State University Wexner Medical Center, Columbus, OH, United States

**Keywords:** exercise, obesity—complications, cardiovascular, type 2 diabetes, myokines

## Abstract

Obesity is a complex disease that affects whole body metabolism and is associated with an increased risk of cardiovascular disease (CVD) and Type 2 diabetes (T2D). Physical exercise results in numerous health benefits and is an important tool to combat obesity and its co-morbidities, including cardiovascular disease. Exercise prevents both the onset and development of cardiovascular disease and is an important therapeutic tool to improve outcomes for patients with cardiovascular disease. Some benefits of exercise include enhanced mitochondrial function, restoration and improvement of vasculature, and the release of myokines from skeletal muscle that preserve or augment cardiovascular function. In this review we will discuss the mechanisms through which exercise promotes cardiovascular health.

## Introduction

Obesity and its associated co-morbidities are increasing at rapid rates across the United States and worldwide (1). Obesity is associated with many adverse health effects, including increased risks of cardiovascular disease (CVD), type 2 diabetes (T2D), certain cancers, and death (2–6). As obesity rates continue to rise, the prevalence of associated comorbidities including T2D and CVD increase concomitantly (7); overweight people are twice as likely, and severely obese people are ten times more likely to develop cardiovascular diseases than individuals of a healthy weight (8).

Regular physical exercise has several beneficial effects on overall health. While decreasing body mass and adiposity are not the primary outcomes of exercise, exercise can mediate several diseases that accompany obesity including T2D and CVD (9–14). Several recent studies have shown that sustained physical activity is associated with decreased markers of inflammation, improved metabolic health, decreased risk of heart failure, and improved overall survival (15–17). Exercise improves overall metabolic health and reduces the development of T2D (18) by improving glucose tolerance (19), insulin sensitivity (20), and decreasing circulating lipid concentrations (21). This occurs primarily through adaptations to the skeletal muscle, liver, and adipose tissue (16, 22, 23). Physical exercise can also improve cardiovascular function through adaptations to the heart and vascular system (17, 24–27). Regular physical exercise decreases resting heart rate, blood pressure, and atherogenic markers, and increases physiological cardiac hypertrophy (13–15, 28). Exercise improves myocardial perfusion and increases high-density lipoprotein (HDL) cholesterol levels, all of which reduce stress on the heart and improve cardiovascular function in healthy and diseased individuals (11, 15, 29, 30). Given the increasing interest in exercise-based therapies, we will discuss the benefits of exercise on cardiovascular health and the potential mechanisms through which they occur.

## Cardiovascular Disease

Cardiovascular disease (CVD) is the leading cause of morbidity and mortality worldwide (31, 32). Almost half of all adults in the United States have at least one key risk factor for development of CVD (i.e., high blood pressure, high cholesterol, or smoking) (33). CVD encompasses a wide range of conditions that affect the heart and vasculature including arrhythmias, dilated, hypertrophic, or idiopathic cardiomyopathies, heart failure and atherosclerosis (34, 35). These conditions can lead to potentially fatal cardiac events such as stroke, myocardial infarction (heart attack), or cardiac arrest (31, 36). Thus, determining various therapeutic tools to prevent or reduce the incidence of CVD is vital.

Although cardiovascular disease can arise in response to multiple factors, the prevalence of obesity-related CVD is rapidly increasing (8). This can occur for several reasons, one being that a high fat diet or obesity can lead to hypertension. In obesity, angiotensin II and aldosterone secretion from abdominal subcutaneous adipose tissue drives activation of the renin-angiotensin system (37–41). Angiotensin II induces vasoconstriction in arterioles, causing arteriolar resistance and increased systemic blood pressure, in addition to stimulating the release of anti-diuretic hormone, which increases water reabsorption in the kidneys. Aldosterone increases the reabsorption of water and sodium into the blood, resulting in increased extracellular fluid volume, thus increasing blood pressure. The renin-angiotensin system also affects the sympathetic nervous system through inhibition of norepinephrine reuptake in the pre-synaptic sympathetic nerve terminals, increasing resting norepinephrine concentration (42), which can cause an increased resting heart rate and eventually development of hypertension (43, 44). Therefore, the renin-angiotensin system and sympathetic nervous system create a positive feedback loop to increase hypertension in obese individuals (40).

Sustained hypertension increases left ventricular afterload, forcing the left ventricle to work harder (45). This leads to pathologic hypertrophy of the ventricular walls and ventricular chamber dilation, eventually culminating in decreased myocardial function and the onset of heart failure (46, 47). As myocardial function declines, the cardiovascular system becomes impaired, resulting in insufficient blood flow. Oxygen and nutrients are then unable to meet the physiological demands of the body, resulting in tachycardia and extreme fatigue, as well as compounding health issues such as pulmonary congestion, fluid retention, and arrhythmias (48, 49).

Another potential cause of obesity-related CVD is metabolic overload of the heart, which can occur independent of hypertension. The heart is a “metabolic omnivore” (50), but in the obesogenic state, and particularly with insulin resistance, fatty acid uptake and utilization is significantly increased (51). This can lead to inefficient β-oxidation and intramyocardial lipid accumulation (52). Because the heart has limited storage capacity, abundant accumulation of excess lipids and toxic lipid metabolites results in “lipotoxicity” which contributes to cardiac dysfunction (53–55). Indeed, several studies have demonstrated that metabolic changes precede structural changes in the heart (56, 57). Cardiac metabolism is also altered in T2D patients who are not obese. As in obesity, T2D is associated with elevated circulating free fatty acids, increased myocardial fatty acid uptake and utilization, and myocardial insulin resistance leading to decreased glucose uptake and utilization in the heart (58–61).

Atherosclerosis is the most common form of CVD, and the development of atherosclerosis progresses slowly in response to persistent exposure to an unhealthy, sedentary lifestyle, including obesity (34, 62). In an obese state, circulating levels of triglycerides and LDL cholesterol are increased (63), causing small plaques to form under endothelial cells of the innermost surface of artery walls (34, 62, 64). While normal endothelial cells can prevent adhesion of these plaques by leukocytes, under obese conditions LDL molecules are oxidized causing endothelial cells to instead express adhesion molecules and chemoattractants (65–67). In response, macrophages take up oxidized LDL and are transformed into foam cells (64, 65) which localize to the fatty plaques within arteries and secrete factors that further promote plaque formation (67, 68). Resulting plaques cause vessel walls to thicken and stiffen, inhibiting blood flow (69). If the plaques become large enough or thrombosis occurs, the inhibition of blood flow can lead to ischemic conditions and cardiac events including stroke, myocardial infarction (MI), or cardiac arrest (70), all of which can be fatal.

## Exercise Training Improves Cardiovascular Health

There are several risk factors leading to the development and progression of CVD, but one of the most prominent is a sedentary lifestyle (34, 35, 71). A sedentary lifestyle can be characterized by both obesity and consistently low levels of physical activity. Thus, lifestyle interventions that aim to increase physical activity and decrease obesity are attractive therapeutic methods to combat most non-congenital types of CVD.

### Physical Activity Decreases Cardiovascular Risk Factors

Regular physical exercise is associated with numerous health benefits to reduce the progression and development of obesity, T2D, and CVD (9–14). Several randomized clinical trials have demonstrated that lifestyle interventions including moderate exercise and a healthy diet improve cardiovascular health in at-risk populations (72, 73). Individuals with metabolic syndrome who participated in a 4 month program of either a diet (caloric restriction) or exercise intervention had reduced adiposity, decreased systolic, diastolic and mean arterial blood pressure, and lower total and low-density lipoprotein (LDL) cholesterol lipid profiles compared to the control group (12). Both the diet and exercise intervention improve these cardiovascular outcomes to a similar extent (74).

Several previous studies have investigated the effects of diet and exercise, independently or in combination, on metabolic and cardiovascular health and have determined that diet, exercise, or a combination of diet and exercise induces weight loss, decreases visceral adiposity, lowers plasma triglycerides, plasma glucose, HDL levels, and blood pressure, and improves VO_2max_ (75–78). Importantly, several of these beneficial effects of exercise are evident independent of weight loss (79). Studies have shown that exercise can improve metabolic and cardiovascular health independent of changes in body weight, including improved glucose homeostasis (80, 81), endothelial function (82), blood pressure (83), and HDL levels (84, 85). These data indicate exercise, independent of changes in body mass, results in significant improvements in cardiovascular and metabolic health. Although a detailed analysis of the vast impact of diet on cardiometabolic health is outside the scope of this review, the importance of diet and exercise in tandem should not be ignored, as many studies have shown that cardiometabolic health is improved to a higher extent in response to a combined diet and exercise programs compared to either intervention alone (86–89).

Exercise has a similar effect on cardiovascular improvements in lean and overweight normoglycemic subjects. In a 1 year study of non-obese individuals, a 16–20% increase in energy expenditure (of any form of exercise) with no diet intervention resulted in a 22.3% decrease in body fat mass and reduced LDL cholesterol, total cholesterol/HDL ratio, and C-reactive protein concentrations, all risk factors associated with CVD (74). In overweight individuals, 7–9 months of low-intensity exercise (walking ~19 km per week at 40–55% VO_2_peak) significantly increased cardiorespiratory fitness compared to sedentary individuals (90). Together these data indicate that exercise interventions decrease the risk or severity of CVD in subjects who are lean, obese, or have type 2 diabetes (12, 74, 90).

### Physical Activity Improves Cardiovascular Function in Patients With CVD

Exercise is also an important therapeutic treatment for patients who have cardiovascular diseases (14). A systematic review of 63 studies found that exercise-based cardiac rehabilitation improved cardiovascular function (91). These studies consisted of various forms of aerobic exercise at a range of intensities (from 50 to 95% VO_2_), over a multitude of time periods (1–47 months). Overall, exercise significantly reduced CVD-related mortality, decreased risk of MI, and improved quality of life (91). Another study looked specifically in patients with atherosclerosis post-revascularization surgery. Patients who underwent 60 min of exercise per day on a cycle ergometer for 4 weeks had an increase blood flow reserve (29%) and improved endothelium-dependent vasodilatation (10). A recent study provided personalized aerobic exercise rehabilitation programs for patients who had an acute myocardial infarction for 1 year after a coronary intervention surgery (92). The patients who underwent the exercise rehabilitation program had increased ejection fraction (60.81 vs. 53% control group), increased exercise tolerance, and reduced cardiovascular risk factors 6 months after starting the exercise rehabilitation program (92). This improvement in cardiovascular health in patients with atherosclerosis or post-MI is likely the result of increased myocardial perfusion in response to exercise, however more research is required to fully understand these mechanisms (10).

One defining characteristic of heart failure is exercise intolerance (93), which resulted in a prescription of bed rest for these patients until the 1950s (94). However, it has now been shown that a monitored rehabilitation program using moderate intensity exercise is safe for heart failure patients, and this has now become an important therapeutic for patients with heart failure (95–97). Meta-analyses and systemic reviews have shown that exercise training in heart failure patients is associated with improved quality of life, reduced risk of hospitalization and decreased rates of long-term mortality (93, 98–102). One study of heart failure patients found that aerobic exercise (walking or cycling) at 60–70% of heart rate reserve 3–5 times per week for over 3 years led to improved health and overall quality of life (determined by a self-reported Kansas City Cardiomyopathy Questionnaire, a 23-question disease-specific questionnaire) (103). Other studies have shown that exercise-based rehabilitation at a moderate intensity in heart failure patients improves cardiorespiratory fitness and increases both exercise endurance capacity and VO_2_max (12–31% increase) (101, 104).

More recent studies have examined the effects of high-intensity exercise on patients with heart failure. A recent study found that 12 weeks of high intensity interval training (HIIT) in heart failure patients (with reduced ejection fraction) was well-tolerated and had similar benefits compared to patients who underwent moderate continuous exercise (MCE) training, including improved left ventricular remodeling and aerobic capacity (105). A separate study found that 4 weeks of HIIT in heart failure patients with preserved ejection fraction improved VO_2peak_ and reduced diastolic dysfunction compared to both pre-training values and compared to the MCE group (78). These studies indicate that both moderate and high intensity exercise training improve cardiovascular function in heart failure patients, likely related to increased endothelium-dependent vasodilation (106) and improved aerobic capacity (78, 101, 105).

### Mechanisms Regulating Exercise-Induced Benefits on Cardiovascular Health

**Figure 1:**
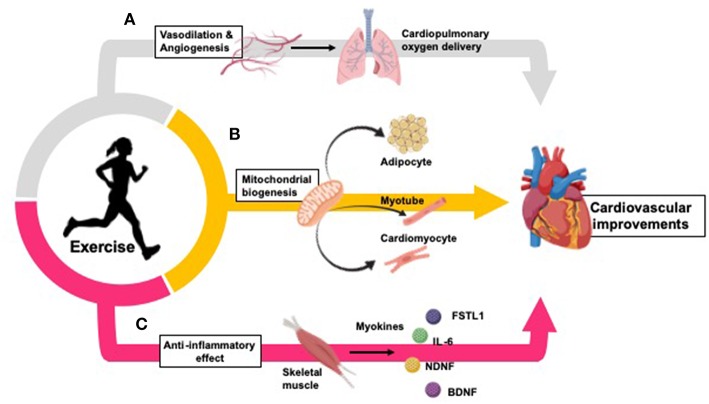
Exercise improves cardiovascular health by inducing changes in oxygen delivery, vasculature, peripheral tissues, and inflammation. **(A)** Exercise improves oxygen delivery throughout the body through promotion of vasodilation and angiogenesis (107–110). **(B)** Exercise increases mitochondrial biogenesis in adipocytes (104, 111, 112), skeletal muscle myotubes (113), and cardiomyocytes (14, 114, 115). **(C)** Exercise causes a long-term anti-inflammatory effect (which is inversely related to the increased inflammation typically seen in CVD and obesity) (116). Myokines released from skeletal muscle during physical exercise partially mediate these anti-inflammatory effects, and promote inter-tissue cross talk to mediate further cardiovascular benefits (117–120).

Multiple mechanisms mediate the benefits of regular physical exercise on cardiovascular health (13, 14) (Figure 1). Exercise represents a major challenge to whole-body homeostasis, and provokes widespread changes in numerous cells, tissues, and organs in response to the increased metabolic demand (121), including adaptations to the cardiovascular system (13, 14).

Exercise induces adaptations in several cell types and tissues throughout the body. Exercise increases mitochondrial biogenesis in adipocytes (104, 111, 112), skeletal muscle myocytes (113), and cardiomyocytes (14, 114, 115), increasing aerobic respiration within these tissues. Additionally, exercise improves oxygen delivery throughout the body through vasodilation and angiogenesis (107–110), protecting against ischemia-reperfusion injury in the heart (122, 123). Further, exercise causes a long-term anti-inflammatory effect which is inversely related to the increased inflammation typically seen in CVD and obesity (116). Myokines released from skeletal muscle during physical exercise partially mediate these anti-inflammatory effects, and promote inter-tissue cross talk to mediate further cardiovascular benefits (117–120).

### Exercise Improves Mitochondrial Biogenesis and Function

Many of the benefits sustained by exercise are due to mitochondrial adaptations throughout the body. For example, exercise improves long-term cardiorespiratory fitness (VO_2_) by increasing the mitochondrial content and desaturation of myoglobin in skeletal muscle tissue, improving the oxidative capacity of skeletal muscle (113, 124, 125). The increase of oxygen uptake and utilization by skeletal muscle (as indicated by arteriovenous oxygen difference; a-vO_2_) in response to regular exercise (126) is protective against a decrease in obesity-related a-vO2, resulting in individuals to require more blood to receive the same amount of oxygen (127).

Mitochondrial biogenesis is augmented in cardiomyocytes in response to exercise (14, 114, 115, 128). This is likely due to enhanced activation of AMP-activated protein kinase (AMPK) and subsequent increase mitochondrial PGC-1α expression (109, 114) Exercise also increases the ability of mitochondria to oxidize fatty acids (the predominant substrate utilized in healthy myocardium), thus increasing the capacity for ATP synthesis (14, 129–133). These exercise-induced enhancements of mitochondrial function are important in preventing cardiovascular dysfunctions often caused by obesity.

Obesity is associated with defective mitochondrial biogenesis in the myocardium (134) and reduced mitochondrial capacity for oxidative phosphorylation and ATP synthesis (135, 136). In heart failure, fatty acid uptake, and utilization is decreased (137), likely causing the heart failure associated shift toward glucose metabolism in order to preserve cardiovascular function (130, 137, 138). However, in advanced heart failure, diabetes, or obesity, myocardial insulin resistance may develop, impairing glucose uptake and accelerating cardiovascular dysfunction (139–141). Importantly, insulin sensitivity is improved in response to regular exercise (142) which is vital in reducing the risk of obesity-related insulin resistance. Insulin has also been indicated to directly regulate mitochondrial metabolism by promoting induction of OPA1, a GTPase that controls mitochondrial cristae integrity, energetics and mitochondrial DNA maintenance (143, 144), thus indicating another potential mechanism of exercise-induced improvements in cardiovascular health through mitochondrial function enhancement.

Reactive oxygen species (ROS) are physiological byproducts of aerobic mitochondrial metabolism and while necessary for initiating cellular repair or apoptosis, increased levels of ROS are associated with inflammation and several forms of CVD (145). While exercise increases the direct production of ROS by mitochondria, the net cellular ROS load is reduced by exercise due to increased action of antioxidant systems (146). Essentially, exercise creates a system in which cells exhibit a “favorable” response within low exposures of ROS, allowing antioxidant systems to work effectively (147).

By increasing the ability of mitochondria to prevent oxidative damage, exercise-induced modifications to mitochondria protect against ischemia-reperfusion damage to the heart. During ischemia, the absence of oxygen from the heart creates an environment in which the return of oxygenated blood flow leads to the induction of inflammation and oxidative stress rather than restoration of normal function (148). In contrast, exercise-induced adaptations to cardiomyocyte mitochondria dampen oxidative damage caused by ischemia-reperfusion, resulting in reduced cardiac injury and decreasing the risk of ischemia-related cardiac dysfunction or death (149–151).

### Exercise Improves Vasculature and Myocardial Perfusion

Exercise training induces vascular adaptations to several tissues (107, 108). In the heart, the increase in vascularization protects against vascular stress and reduces the likelihood of a cardiac event (24–26). These adaptations are mediated through increased expression of vascular endothelial nitric oxide synthase (eNOS). Exercise increases the intensity of physiological shear stress, inducing the shear stress-dependent activity of c-Src in endothelial cells and increasing expression of eNOS (27, 152). In the vascular endothelium, eNOS catalyzes the production of nitric oxide (NO) which causes vasodilation, inhibits platelet aggregation and prevents leukocyte adhesion to vessel walls, thus reducing the onset of atherosclerosis, thrombosis, ischemia, or other cardiac events (152, 153).

Exercise also induces angiogenesis, however the mechanisms regulating this process are unclear. It has been hypothesized that the increase in nitric oxide (NO) production after exercise upregulates pro-angiogenic factors, particularly vascular endothelial growth factor (VEGF) (154). One recent study determined that male rats who underwent exercise training for 10 weeks after MI had increased Akt phosphorylation of eNOS, and reactivation of cardiac VEGF pathway activity, resulting in increased angiogenesis (155). While the mechanisms are not completely defined, it is clear that exercise induces arteriogenesis, increases angiogenesis and protects against vascular stress, thus decreasing the possibility of a cardiac event (107–110, 122, 123).

### Exercise Reduces Chronic Inflammation

Inflammation is a complex yet normal biological reaction to damaging stimuli (156). Chronic inflammation is associated with multiple diseases including obesity, T2D, and CVD (116, 157). Excess consumption of nutrients causes cells including adipocytes (158), hepatocytes (159), islet cells (160), and skeletal muscle cells (161) to activate the transcription factors nuclear factor kappa-light-chain-enhancer of activated B cells (NF-κB) and activator protein 1 (AP-1), increase expression of toll-like receptor 4 (TLR4) (162, 163), and stimulate the release of cytokines such as TNF-α, IL-6, IL-1β, and CCL2 (158, 164). The subsequent inflammation is modest in comparison to inflammatory responses during infection or injury (165) but remains as a chronic response to obesity termed “meta-inflammation” (163). Exercise, however, results in a long-term anti-inflammatory effect (116, 156). The exercise-induced reduction of meta-inflammation during disease is hypothesized by some to be related to downregulation of NF-κB (166–168), but exercise also decreases monocyte accumulation and suppresses the release of TNF-α and other pro-inflammatory adipokines, creating an anti-inflammatory effect (169–172).

Excess immune activation caused by obesity is of particular concern for vascular health, as activation of TLR4 causes monocyte recruitment and conversion to foam cells, driving the progression of atherosclerosis (67, 173). Exercise prevents the development of atherosclerosis by reducing expression of TLRs on monocytes and macrophages, which subsequently decreases the availability of TLR4 ligands and inhibiting pro-inflammatory cytokine production (170, 171, 174). Exercise also decreases pro-inflammatory N-terminal pro b-type natriuretic (NT-proBNP) and high-sensitivity C-reactive protein (hsCRP) within the heart, both of which are predictors of heart failure in atherosclerosis (175, 176).

### Exercise Enhances Inter-tissue Communication Through Release of Myokines

**Figure 2:**
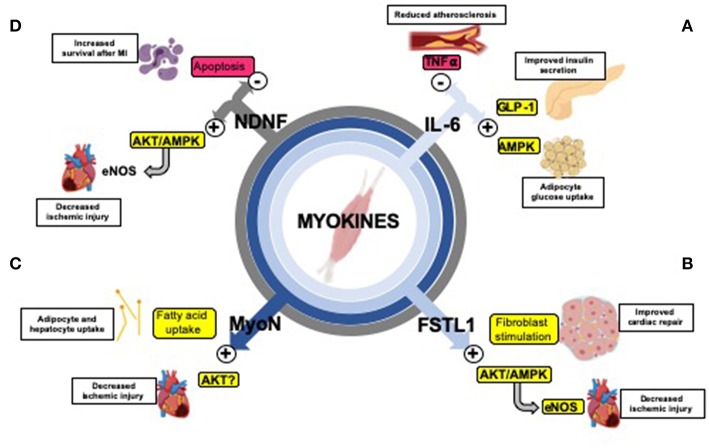
Exercise-induced myokines mediate organ cross-talk and improve cardiometabolic health. **(A)** The myokine IL-6 inhibits TNF-α (186), reducing inflammation and protecting against the formation of atherosclerosis (187); stimulates GLP-1 secretion causing improved insulin secretion (188); increases lipolysis and fatty acid oxidation in adipose tissue (189) and increases glucose uptake through the AMPK signaling pathway (190, 191). **(B)** Fstl1 decreases ischemic injury size through activation of the Akt/AMPK pathway (activating eNOS and enhancing revascularization) (118, 119) and early fibroblast stimulation (which aids in repair after ischemia-reperfusion) (192). **(C)** Myonectin (MyoN) increases fatty acid uptake in adipocytes and hepatocytes (117), and promotes protects against ischemic injury in the heart, possibly through Akt activation (193). **(D)** NDNF improves survival after myocardial infarction (MI) by reducing apoptosis (120) through stimulation of the Akt/AMPK/eNOS pathway (enhancing revascularization) (194).

Skeletal muscle can act as a secretory organ by stimulating the production, secretion, and expression of specific myokines after contraction (177–179). Myokines are chemical messengers that function in an autocrine, paracrine, or endocrine manner to influence crosstalk between different organs including skeletal muscle, liver, and adipose tissue (180–185). They are of great interest with regards to cardiovascular health because the well-known protective actions of exercise on cardiovascular function are at least partially mediated by increased secretion of myokines (Figure 2) (195). Some myokines that impact cardiovascular health include IL-6, myonectin, Fstl1, and NDNF (196).

#### Interleukin-6 (IL-6)

IL-6 was introduced as the first myokine over a decade ago (197). Circulating levels of IL-6 are increased in response an acute bout of aerobic exercise (198, 199) and can act in an endocrine fashion to improve metabolic and cardiovascular health. Exercise-induced elevated concentrations of IL-6 can stimulate glucagon-like peptide-1 (GLP-1) secretion from intestinal L cells and pancreatic α cells, leading to improvements in insulin secretion and glycemia (188). IL-6 also increases lipolysis and fatty acid oxidation in adipose tissue (189) and can increase glucose uptake through stimulation of the AMP-activated protein kinase (AMPK) signaling pathway (190, 191). With regard to cardiovascular function, IL-6 can reduce inflammation by inhibiting tumor necrosis factor-α (TNF- α) (186). This results in a protective effect on cardiovascular health because TNF- α is involved in the formation of atherosclerosis, development of heart failure, and subsequent complications, including myocardial infarction (MI) (187). More investigation is required to determine the direct effects of IL-6 action on cardiovascular health.

#### Myonectin

Myonectin (or CTRP15) is abundantly expressed in skeletal muscle and is increased in response to chronic aerobic exercise (117). Importantly, injection of myonectin into wild-type mice decreases circulating free fatty acids levels by increasing fatty acid uptake in adipocytes and hepatocytes (117). Myonectin has also been identified to have protective effects on cardiovascular health; mice deficient in Myonectin had enhanced ischemic injury in response to MI while systemic delivery of myonectin attenuated ischemic injury (200). Further work is needed to determine whether these benefits are observed in response to an increase in myonectin after exercise.

#### Follistatin-Like 1 (Fstl1)

Fstl1, also referred to as TSC-36, is a secreted glycoprotein that belongs to the follistatin family of proteins and is upregulated in skeletal muscle in response to exercise (194, 201, 202). Expression of Fstl1 is also increased in ischemic and hypertrophic hearts of mice and functions in a protective manner (118). Systemic administration of Fstl1 in both mouse and swine models led to reduced apoptosis, inflammation and injury size following ischemia-reperfusion (118, 119). *In vitro*, treatment of cultured cardiomyocytes with Fstl1 reduces apoptosis in response to hypoxia-reoxygenation by activating Akt and AMPK (118, 119). One recent study demonstrated that Fstl1 stimulates early fibroblast activation, which is required for acute repair and protects the heart from rupture after ischemia-reperfusion (192). While the exact role of an exercise-induced increase in Flst1 on cardiovascular function has not been defined, these data indicate that Fstl1 is increased in response to exercise, and an increase in circulating Fstl1 functions to repair cardiovascular damage and improve cardiovascular function (202).

#### Neuron-Derived Neurotrophic Factor (NDNF)

NDNF is a glycosylated protein secreted from the endothelial cells of skeletal muscle (203). Although initially identified as a neurotrophic factor expressed in mouse brain and spinal cord (204), NDNF is also released from skeletal muscle in response to exercise (203) and acts as a hypoxia-induced pro-angiogenic factor that stimulates endothelial cell network formation through activation of the Akt/eNOS signaling pathway (194). This pro-angiogenic affect is an important component in the recovery from MI; intramuscular administration of NDNF using an adenoviral vector improved systolic function in a mouse model after MI (120). Increased NDNF levels are also associated with reduced myocardial hypertrophy and apoptosis in post-MI hearts (120). Another study showed that down-regulation of NDNF by siRNA impairs recovery from ischemia-reperfusion injury (205). Treatment of NDNF in cardiomyocytes also reduces hypoxia-induced apoptosis via activation of the focal adhesion kinase/Akt-dependent pathway (120). Additionally, increased levels of NDNF released from skeletal muscle in response to exercise enhance fatty acid oxidation through activation of AMPK (203). These data demonstrate the importance of NDNF as an endogenous ischemia- and exercise inducible factor that can enhance revascularization and therefore have a cardiovascular protective effect.

## Conclusions

The rate of obesity-related cardiovascular disease is rapidly increasing, and often associated with additional co-morbidities including type 2 diabetes (3, 6, 8). It is clear that exercise reduces cardiovascular risk factors, and this reduction in risk factors is independent of changes to body weight or incidence of type 2 diabetes (75–77, 79, 206, 207). Exercise is also an important therapeutic treatment for patients who have cardiovascular diseases (14), further demonstrating the protective and restorative properties of exercise. In patients with CVD, exercise improved endothelium-dependent vasodilatation, increased ejection fraction and exercise tolerance, improved quality of life, and reduced CVD-related mortality (10, 91, 92, 101, 103, 208–211). Exercise improves cardiovascular health by several mechanisms including increased mitochondrial biogenesis and fatty acid oxidation (14, 114, 115, 128–130) dilation of blood vessels causing improved myocardial perfusion (9–11), and reduction of inflammation providing protection against the development of atherosclerosis (67, 116, 156). Myokines released from skeletal muscle during exercise also mediate systemic and cardiovascular health benefits through an anti-inflammatory action, increased fatty acid oxidation, increased glucose uptake, and improved insulin secretion and sensitivity (117, 186, 193, 196, 212–214). Importantly, several myokines (IL-6, Myonectin, Fstl1, and NDNF) have also been shown to have cardiovascular protective effects in response to ischemia-reperfusion injury (117–120, 186, 187).

While it is clear that exercise is important, the mechanistic pathways behind exercise-induced benefits on cardiovascular health are still being identified. Further understanding of the molecular mechanisms through which exercise improves cardiovascular function will lead to the development of therapeutics which can act in conjunction with exercise programs, and for individuals whom are unable or unwilling to exercise to amplify the beneficial effects of exercise.

Future research will investigate the effects of cardiac specific proteins on cardiovascular health, expanding research into the areas of system cross-talk will help delineate how other tissues, skeletal muscle in particular, can mediate cardiovascular improvements via myokine release. How these myokines affect cardiovascular function, including adaptations to mitochondrial activity, angiogenesis and inflammatory responses will provide insight into new mechanisms for the beneficial effects of exercise on cardiovascular function. Accordingly, myokines may act as potential targets for heart disease prevention and therapies. Recent studies have investigated the use of gene therapies, including the use of adeno-associated virus, on cardiovascular function. While these therapies have not been fully optimized with remaining issues in immunogenicity, efficacy and genotoxicity (215), their development provides excitement for the potential therapies focused on exercise-induced myokines that improve cardiovascular function as a treatment for patients who are unable, or perhaps unwilling, to exercise. Together these data highlight the importance of exercise and exercise-related therapies to both prevents the development of cardiovascular disease and promotes recovery and improved health in patients with CVD.

## Author Contributions

KP, KKB, and KIS outlined, drafted, and contributed to the writing of the manuscript. All authors approved the final version of the manuscript.

### Conflict of Interest Statement

The authors declare that the research was conducted in the absence of any commercial or financial relationships that could be construed as a potential conflict of interest.
